# From habit to high risk: The influence of multidimensional lifestyle changes on internet addiction risk among junior high school students and its predictive utility

**DOI:** 10.1371/journal.pone.0345506

**Published:** 2026-03-25

**Authors:** Xinwei Yu, Li Zhang, Xuejian Su, Ye Yu, Bo Liu, Lifang Zhou, Xiaopeng Deng

**Affiliations:** 1 Health Science Center, Yangtze University, Jingzhou City, Hubei Province, China; 2 Mental Health Center of Yangtze University, Jingzhou City, Hubei Province, China; 3 Jingzhou Mental Health Center, Jingzhou City, Hubei Province, China; 4 Jingzhou Rongjun Special Care Hospital, Jingzhou City, Hubei Province, China; Johns Hopkins Bloomberg School of Public Health: Johns Hopkins University Bloomberg School of Public Health, UNITED STATES OF AMERICA

## Abstract

**Objective:**

To investigate how changes in the daily lifestyles of junior high school students influence the risk of Internet addiction, and to develop precise, lifestyle-based intervention strategies for preventing and reducing such addiction.

**Methods:**

In April 2024, a follow-up survey was conducted on a cohort first assessed in September 2023. The relationship between various dynamic changes in daily lifestyle—encompassing four dimensions: exercise, smart device ownership, diet, sleep-wake patterns and the risk of Internet addiction was analyzed. The predictive performance of these factors was evaluated using Receiver Operating Characteristic (ROC) curves.

**Results:**

Of the 10,535 participants enrolled at baseline (T1), 9,750 were successfully followed up at T2, yielding a retention rate of 92.55% (9,750/10,535). Among these, 7,853 provided complete and valid questionnaire data, corresponding to a T2 effective data rate of 80.54% (7,853/9,750). The detected rate of Internet addiction risk increased from 10.62% at T1 to 13.35% at T2. Multivariate analysis identified the following behavioral changes as significant risk factors for Internet addiction: going to bed after 10:00 pm or having delayed sleep onset timing (OR = 2.859, 95% CI: 2.319–3.525); developing late-night eating habit (OR = 1.932, 95% CI: 1.494–2.499); becoming a smart device owner (OR = 1.773, 95% CI: 1.307–2.405); being a non-habitual napper (OR = 1.699, 95% CI: 1.408–2.049); decreased pursuit of dietary balance (OR = 1.654, 95% CI: 1.300–2.104); decreased active exercise (OR = 1.575, 95% CI: 1.222–2.031); and decrease in exercise duration per session (OR = 1.436, 95% CI: 1.117–1.846). A predictive model incorporating six key variables (excluding change in habitual late-night eating) demonstrated acceptable performance, with an area under the ROC curve (AUC) of 0.721 (95% CI: 0.701–0.741).

**Conclusion:**

This six-month longitudinal study systematically reveals that adverse changes across four lifestyle dimensions significantly increase the risk of Internet addiction among junior high school students. These findings provide an empirical basis for developing targeted interventions to prevent and reduce this risk.

## Introduction

Internet addiction (IA) refers to a series of psychological and behavioral problems caused by excessive use of the Internet [1]. With the widespread adoption of the Internet and the rapid growth of its user base, IA has become an increasingly serious public health concern [2]. Adolescence is a critical transitional period for physical and psychological development, during which self-regulation and cognitive control mechanisms are not yet fully mature [3]. The junior high school stage is a golden period for adolescents to cultivate interests, establish aspirations, develop their personalities, and shape their worldview. During this phase, they are more susceptible to developing dependent patterns of Internet use [4,5]. Epidemiological data indicate that IA has become a severe mental health problem among adolescents in many countries, with prevalence rates of 17% in mainland China, 16% in Hong Kong SAR, China, and 10% in South Korea, 14% in Malaysia, and 21% in the Philippines [6–9]. These findings underscore the need for widespread attention and concern.

Traditionally, the concept of habit has been simplified as an automatic “stimulus-response” behavioral pattern, often invoked to explain the repetitive and persistent nature of addictive behaviors [10]. However, this atomistic and mechanistic view of habit falls short in fully explaining how addiction can profoundly reshape an individual’s overall life and world of meaning. In recent years, the emerging theory of enactive cognition offers a more integrative perspective for habit research. This theory conceptualizes habit as an autonomous, unstable, and self-sustaining generative system. This system is dynamically coupled through the interaction of neural, bodily, and environmental processes, and endows the individual with distinct normativity and identity [11]. Crucially, habits do not exist in isolation; rather, they form interdependent, competing, or synergistic networks that collectively constitute an individual’s “form of life” or “local identity” [12].

Within this framework, a maladaptive habit—such as addiction—can be defined as one that, despite possessing an inherent drive for self-maintenance, systematically suppresses the expression of other contextually relevant habits, thereby impairing the individual’s overall well-being and identity integration. Internet addiction exemplifies such a gradually dominant “bad habit.” By contracting the individual’s “field of relevant affordances”—that is, the range of perceived possibilities for action—it marginalizes the expression of habits in other life domains (such as studying, physical exercise, and face-to-face social interaction), ultimately threatening the psychosocial development and physical health of adolescents [13].

Lifestyle, as the embodied expression of clusters of habits, constitutes a crucial context for shaping and sustaining individual health [14]. Key dimensions of a healthy lifestyle include regular physical exercise, balanced dietary intake, scientifically structured sleep-wake routines, and moderated use of smart devices. These dimensions collectively provide a protective foundation against addictive tendencies by enhancing self-regulatory resources and improving psychological resilience [15]. Conversely, negative shifts in these habitual dimensions—such as reduced physical activity, sleep disturbances, and excessive extension of smart device use—can undermine an individual’s capacity for autonomous regulation, thereby creating an ecological niche for the transformation of ordinary internet use into a dominant addictive behavior [16].

Currently, digital devices such as smartphones have become deeply embedded in daily life, and their usage patterns are themselves an integral part of modern lifestyles [17]. As core components of lifestyle, habits—when developed in a positive direction—contribute to the formation of a stable, proactive, and healthy way of living [18,19]. In contrast, maladaptive lifestyles may weaken an individual’s self-control, thereby increasing dependence on the internet and elevating the risk of internet addiction [16,20].

Most existing studies on Internet addiction are cross-sectional, primarily analyzing its current status and associated factors, with limited exploration into the relationship between changes in various daily lifestyle factors and Internet addiction risk among junior high school students. In contrast, this longitudinal study systematically examines the association between dynamic changes in daily lifestyle—across four dimensions: exercise, smart device ownership, diet, sleep-wake patterns and the risk of Internet addiction risk. The findings provide evidence-based insights for preventing and reducing the risk of Internet addiction.

## Subjects and methods

### Study subjects

The recruitment period for this study spanned from September 19 to October 6, 2023 (T1), during which 10,535 mental health questionnaires were collected from all junior high school students in a designated district of Jingzhou City. Follow-up was conducted from March 27 to April 15, 2024 (T2), with 9,750 responses collected from the same cohort. After excluding duplicates, questionnaires with missing key variables (such as name), and those with excessively short response times (less than 2 seconds per item [21]), 7,853 valid paired questionnaires were retained, accounting for 80.54% of T2 respondents and constituting the final analytical sample.

Comparison between attrition cases and the final sample revealed no significant differences in gender (χ² = 2.261, P = 0.133), grade (χ² = 1.806, P = 0.179), ethnicity (χ² = 0.005, P = 0.946), only child or not (χ² = 0.468, P = 0.494), living situation (χ² = 5.485, P = 0.064), mode of school travel (χ² = 0.805, P = 0.369), or IAT score (Z = −0.940, P = 0.347), indicating no structural attrition bias.

The mean age of participants was 13.26 ± 0.92 years at T1 and 13.86 ± 0.92 years at T2. Electronic informed consent was obtained from all students and their parents prior to participation. The study protocol was approved by the Ethics Committee of Jingzhou Mental Health Center (Approval No.: 2021LL0501).

### Study instruments

#### General situation and daily lifestyle survey questionnaire.

Both surveys were conducted using the “Sunshine Adolescent Mental Health Questionnaire [22]” developed by Professor Maosheng Ran of Sichuan University. The questionnaire comprises three sections: personal characteristics (age, gender, grade, ethnicity); family factors (only child or not, living situation, mode of school travel); and the daily lifestyle of junior high school students, covering four dimensions: physical exercise, smart device ownership, diet, and sleep-wake patterns. Specific measurements within these dimensions included exercise willingness (little to no exercise, passive exercise, active exercise), weekly exercise frequency (1–2 times/week, 3–5 times/week, ≥ 6 times/week), exercise duration per session (<0.5 h/session, 0.5–1.0 h/session, > 1.0 h/session), smart device ownership (smart device owner, non-owner of smart device), pursuit of dietary balance (minimal pursuit (≤25%), low pursuit (26–49%), moderate pursuit (50–74%), high pursuit (≥75%)), late-night eating habit (no habitual late-night eating, habitual late-night eating), bedtime (bedtime before 10:00 PM, bedtime after 10:00 PM), and napping habit (no habitual napping, habitual napping).

#### Internet Addiction Test (IAT).

Internet addiction risk was assessed using the Internet Addiction Test [1] (IAT) developed by Young in 1998, which demonstrated a Cronbach’s α coefficient of 0.93 [23] in this study. The IAT comprises 20 items, each rated on a 5-point Likert scale ranging from 1 (“rarely”) to 5 (“always”). The total score ranges from 20 to 100, with higher scores indicating a greater risk of internet addiction [1]. Participants scoring 20–49 were classified as normal internet users, while an IAT score ≥ 50 was defined as indicating internet addiction risk [24,25]. In the present study, the Cronbach’s α coefficients for the IAT were 0.919 at T1 and 0.936 at T2.

## Methods

Data analysis was performed using SPSS 27.0 software. Categorical data are presented as numbers (percentages), and intergroup comparisons of demographic characteristics were conducted using the chi-square (χ²) test. To investigate the impact of lifestyle changes on the risk of Internet addiction among junior high school students, hierarchical regression analysis was performed while controlling for known confounding factors. First, a baseline model (Model 1) was constructed, incorporating variables found to be significant in the chi-square tests. Subsequently, a full model (Model 2) was developed by further adding change scores for daily lifestyle factors—specifically, physical exercise, smart device ownership, diet, and sleep-wake patterns. The goodness of fit between the two models (e.g., changes in R²) was compared, and odds ratios (ORs) for each variable in the full model were reported to more accurately assess the effect of lifestyle changes on Internet addiction.

Receiver Operating Characteristic (ROC) curves were plotted to evaluate the predictive performance of the constructed models. An area under the curve (AUC) greater than 0.70 indicates acceptable predictive performance [26]. The optimal cutoff value was determined using the Youden index, and the DeLong test was applied to compare different ROC curves in order to identify the best predictive model. The reliability of the IAT scores was assessed using Cronbach’s α coefficient, with an acceptable range defined as 0.70 to 0.95 [27]. In this study, a two-sided *P* value < 0.05 was considered statistically significant.

## Results and discussion

### Detection of internet addiction risk in the full cohort

At T1, 834 participants were identified as being at risk for internet addiction, corresponding to a detection rate of 10.62%. Among them, 427 were male (10.49%) and 407 were female (10.76%), with no statistically significant gender difference (*P* > 0.05). By 2024, the number of individuals identified with internet addiction risk increased to 1,048, raising the detection rate to 13.35%. Within this group, 512 were male (12.58%) and 536 were female (14.17%), showing a statistically significant gender difference in 2024 (*P* < 0.05). No significant associations were found between internet addiction risk and only child or not (χ² = 1.136, *P* > 0.05) or ethnicity (χ² = 2.231, *P* > 0.05). However, significant associations were observed with grade (χ² = 43.719, *P* < 0.05), living situation (χ² = 24.269, *P* < 0.05), and mode of school travel (χ² = 18.451, *P* < 0.05). Detailed results are presented in Table 1.

**Table 1:** Demographic Characteristics Across Internet Addiction Status Groups.

Variables	Non-internet addiction group (n = 6385)	Disappearing group (n = 420)	Newly established group (n = 634)	Continuous group (n = 414)	Chi-square value
Grade					43.719^***^
Grade 7	2678 (41.94%)	103 (24.52%)	228 (35.96%)	88 (21.26%)	
Grade 8	2042 (31.98%)	166 (39.52%)	236 (37.22%)	162 (39.13%)	
Grade 9	1665 (26.08%)	151 (35.95%)	170 (26.81%)	164 (39.61%)	
Gender					4.317^*^
Male	3319 (51.98%)	240 (57.14%)	325 (51.26%)	187 (45.17%)	
Female	3066 (48.02%)	180 (42.86%)	309 (48.74%)	227 (54.83%)	
Ethnicity					2.231
Han	6163 (96.52%)	406 (96.67%)	616 (97.16%)	405 (97.83%)	
Other	222 (3.48%)	14 (3.33%)	18 (2.84%)	9 (2.17%)	
Only child or not					1.136
No	3258 (51.03%)	219 (52.14%)	333 (52.52%)	221 (53.38%)	
Yes	3127 (48.97%)	201 (47.86%)	301 (47.48%)	193 (46.62%)	
Living situation					24.269^***^
Living with parents	3867 (60.56%)	226 (53.81%)	359 (56.62%)	199 (48.07%)	
Living with parents and grandparents	1869 (29.27%)	134 (31.90%)	198 (31.23%)	140 (33.82%)	
Divorced family	338 (5.29%)	31 (7.38%)	45 (7.10%)	33 (7.97%)	
Reconstituted family	142 (2.22%)	12 (2.86%)	13 (2.05%)	24 (5.80%)	
Other	169 (2.65%)	17 (4.05%)	19 (3.00%)	18 (4.35%)	
Mode of school travel					18.451^***^
Parents pick up	2942 (46.08%)	161 (38.33%)	264 (41.64%)	140 (33.82%)	
Go to school by oneself	1443 (22.60%)	113 (26.90%)	157 (24.76%)	108 (26.09%)	
Board	2000 (31.32%)	146 (34.76%)	213 (33.60%)	166 (40.10%)	

*p < 0.05, **p < 0.01, ***p < 0.001.

Based on the longitudinal changes in internet addiction status across the two surveys, participants were categorized into four trajectory groups: Non-internet addiction group (IAT scores < 50 at both T1 and T2, n = 6385); Newly established group (IAT score < 50 at T1 and ≥ 50 at T2, n = 634); Disappearing group (IAT score ≥ 50 at T1 and < 50 at T2, n = 420); and Continuous group (IAT scores ≥ 50 at both T1 and T2, n = 414).

**Fig 1:**
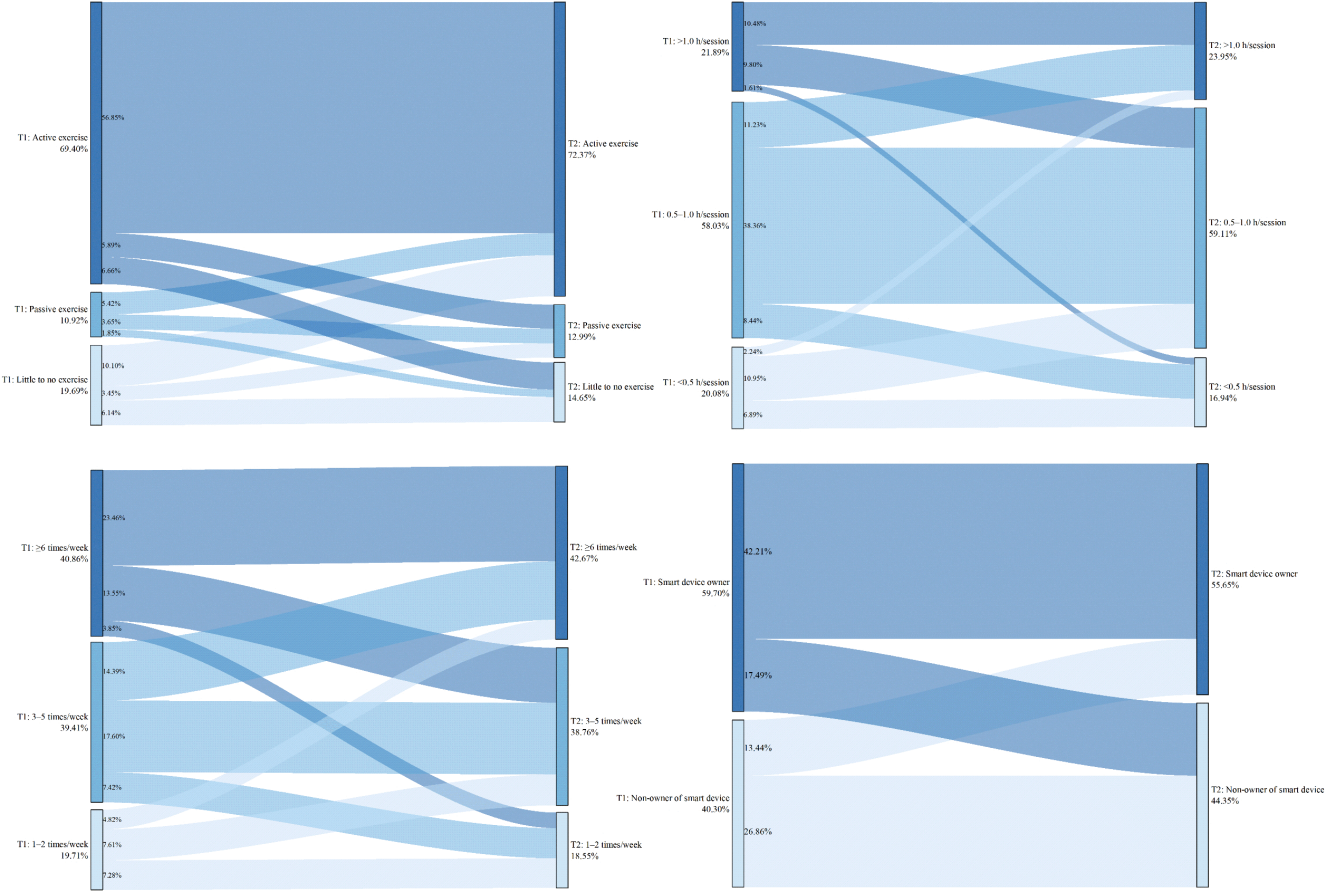
Non-internet addiction group—Transitions in exercise and smart device ownership from T1 to T2.

**Fig 2:**
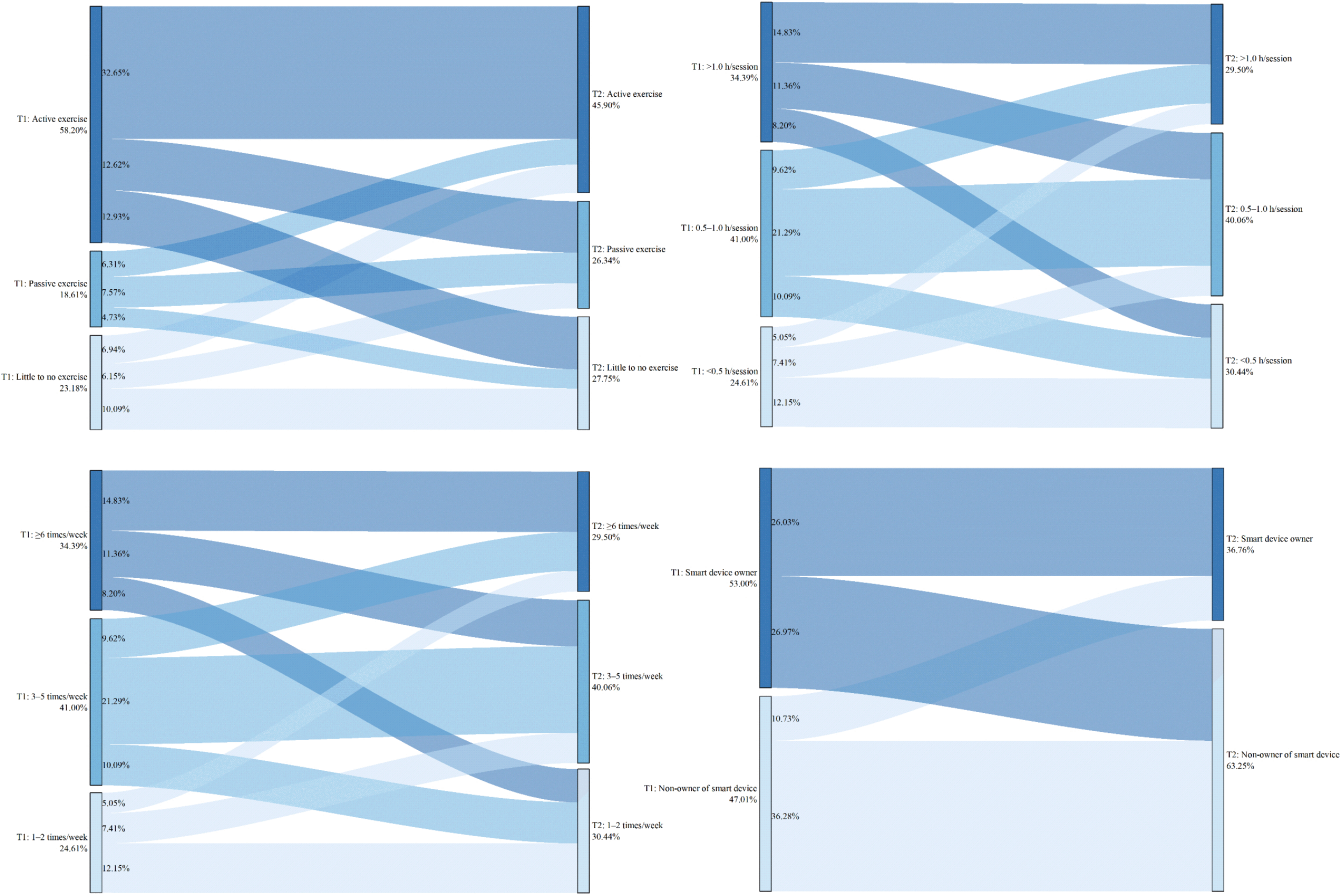
Newly established group—Transitions in exercise and smart device ownership from T1 to T2.

**Fig 3:**
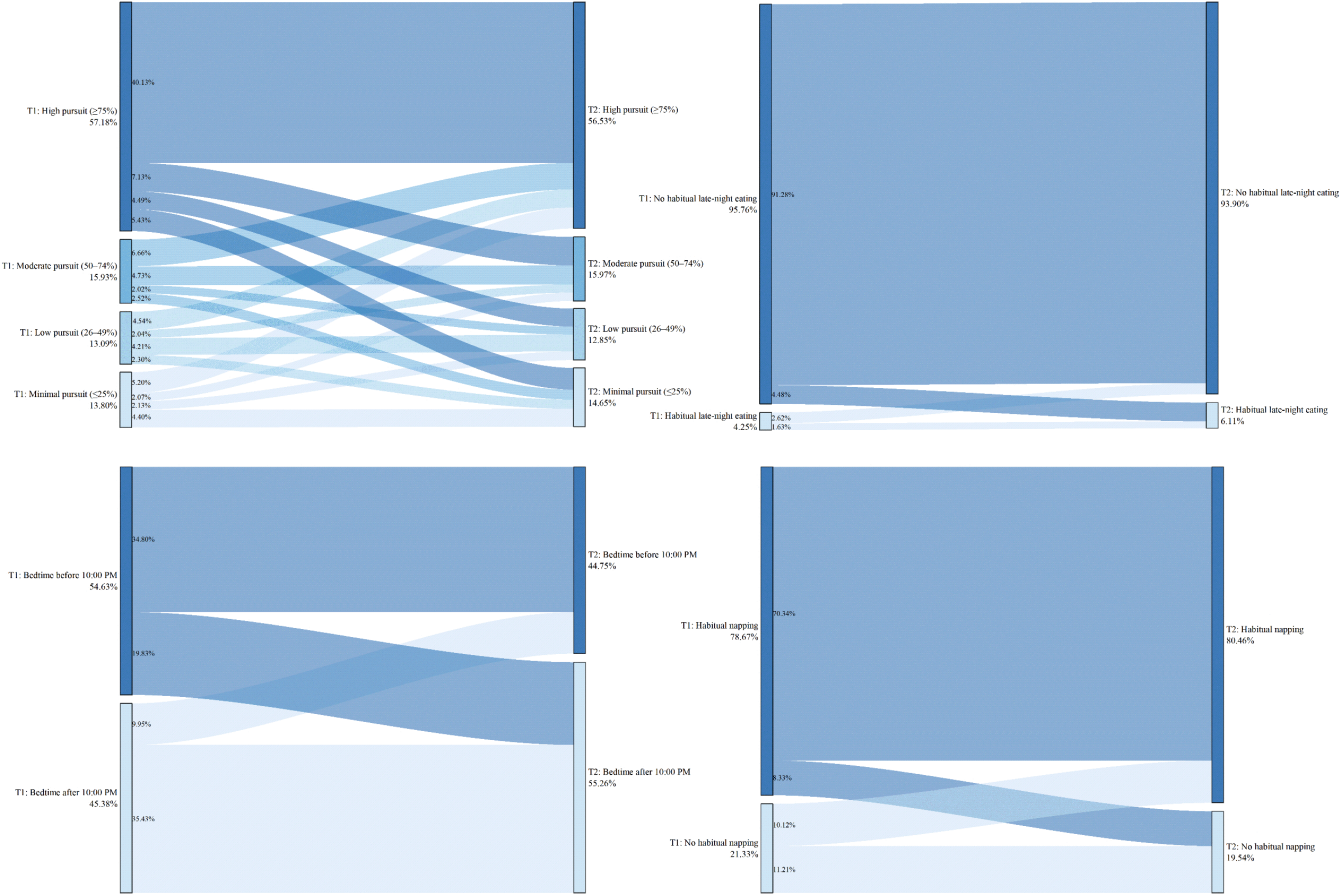
Non-internet addiction group—Transitions in diet and sleep-wake patterns from T1 to T2.

**Fig 4:**
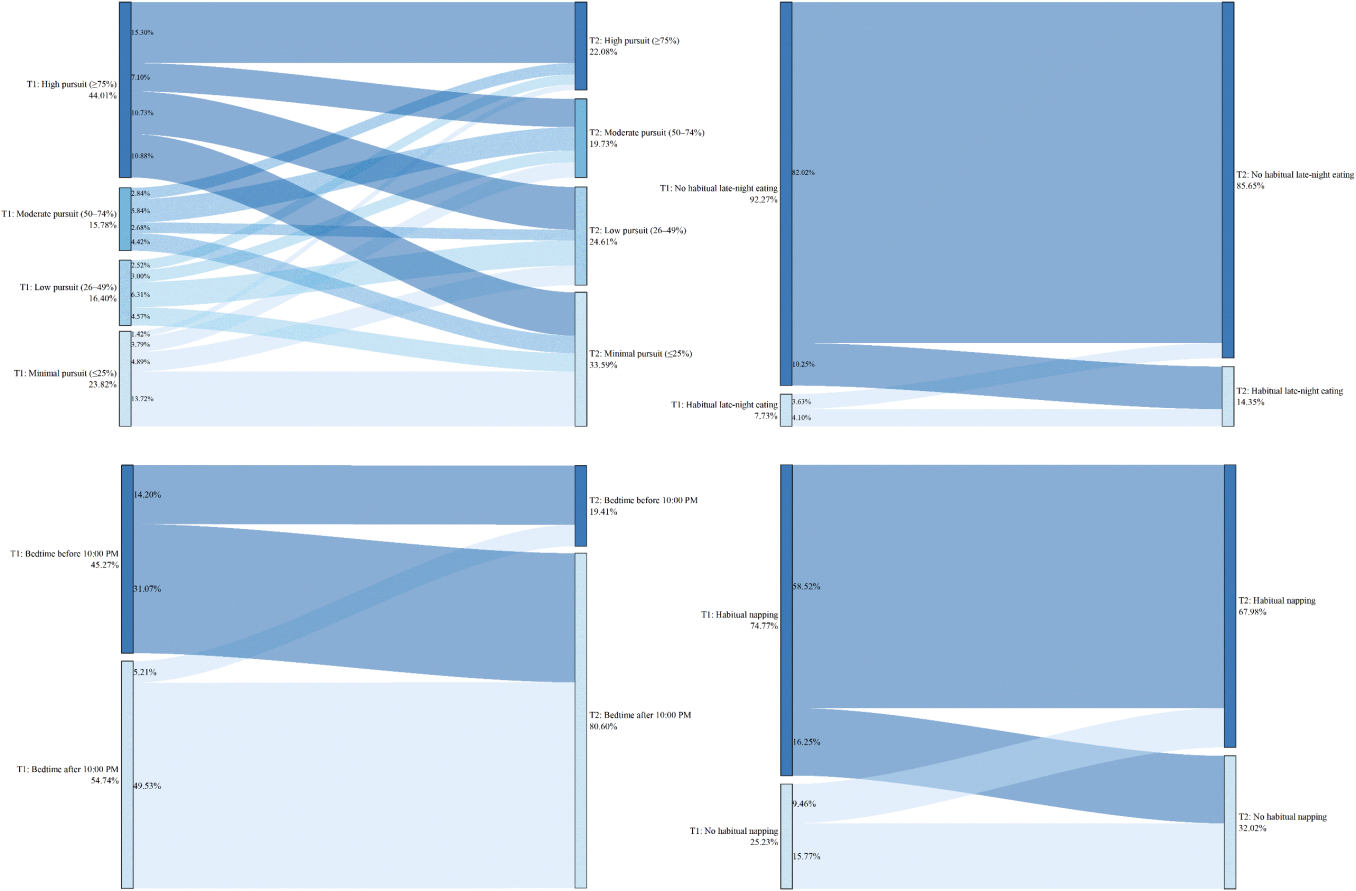
Newly established group—Transitions in diet and sleep-wake patterns from T1 to T2.

**Fig 5:**
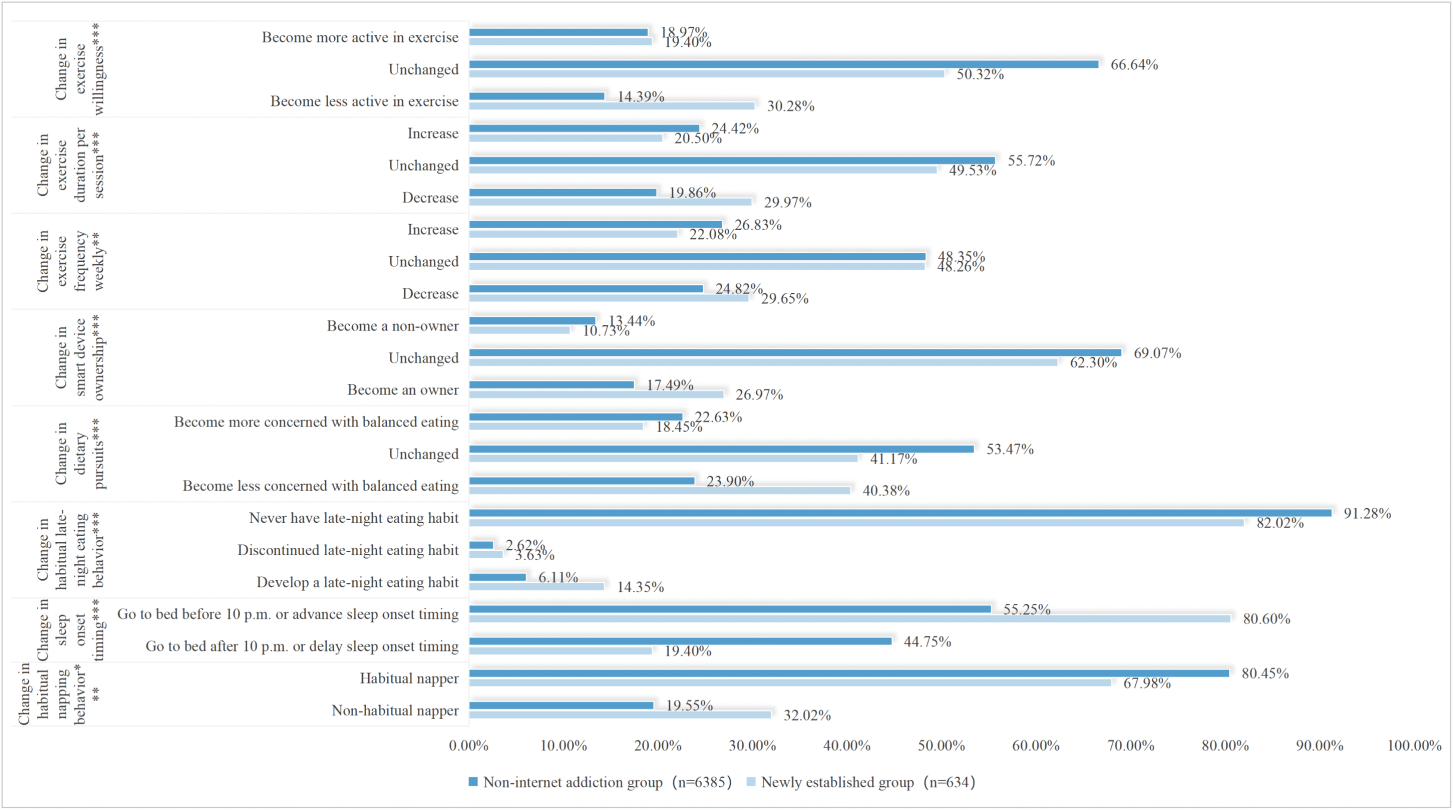
Univariate analysis of variable changes between the non-internet addiction group and Newly established group. *p < 0.05, **p < 0.01, ***p < 0.001.

The above results present the baseline characteristics of the four groups within the full sample. To investigate which specific changes in factors increase the risk of internet addiction, a comparison was made between the Non-internet addiction group and the Newly established group. Upon re-conducting chi-square tests for these two groups, only the variable grade remained significantly different (χ² = 9.986, *P* = 0.007). In contrast, significant associations with group membership were found for the following lifestyle changes: change in dietary pursuits (χ² = 82.878), change in habitual late-night eating behavior (χ² = 64.864), change in habitual napping behavior (χ² = 54.715), change in sleep onset timing (χ² = 151.641), change in exercise willingness (χ² = 115.652), change in exercise frequency weekly (χ² = 10.247), change in exercise duration per session (χ² = 36.059), and change in smart device ownership (χ² = 35.302), with *P* < 0.05 for all. Detailed results are illustrated in Figs 1–5.

### Based on the presence or absence of internet addiction risk, a hierarchical regression analysis was conducted, focusing specifically on the non-internet addiction group and the newly established group

This study employed a scoring method to construct change indicators, quantifying longitudinal changes in relevant behavioral characteristics between the two survey waves. The specific definitions for each variable are as follows:

Change in Ordinal Variables: For ordinal categorical variables, including “bedtime” (1=before 10:00 pm, 2=after 10:00 pm), “weekly exercise frequency,” “exercise duration per session,” and “pursuit of dietary balance” (scored from 1 = minimal to 4 = high), the change score was calculated by subtracting the T1 score from the T2 score. A positive value indicates a shift toward later bedtime, more frequent exercise, longer exercise duration, or higher pursuit of dietary balance; a negative value indicates a change in the opposite direction.

Change in Exercise Willingness: The original response options for exercise willingness were coded as “1=active, 2=passive, 3=little to no exercise.” Similarly, the difference between T2 and T1 scores was calculated to define the “change in exercise willingness”: a positive value indicates increased willingness (i.e., becoming more active), a negative value indicates decreased willingness (i.e., becoming less active), and zero indicates no change.

Change in Other Binary Variables: For binary variables, including “habitual late-night eating,” “habitual napping,” and “smart device ownership” (yes/no), responses were compared directly between the two surveys: a change from “no” to “yes” was defined as “newly established”; a change from “yes” to “no” was defined as “discontinued”; and consistent responses were defined as “no change.”

**Fig 6:**
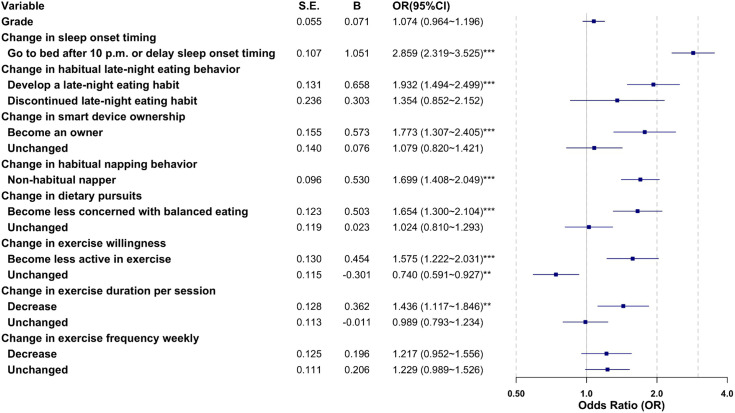
Hierarchical Regression Analysis of Lifestyle Changes on Internet Addiction. Notes: B = Coefficient, S.E. = Standard Error.

The presence or absence of internet addiction risk at T2 was set as the dependent variable (0 = no, 1 = yes). All change scores, along with the demographic variable grade, were included as continuous or categorical predictor variables in a hierarchical regression model. The model fit statistics indicated that Model 1 (Omnibus test: χ² = 3.967, P = 0.046) had poor fit. After incorporating the lifestyle change variables, Model 2 (Omnibus test: χ² = 390.192, P < 0.001) showed a significant improvement in fit, with noticeable increases in both Cox & Snell R² and Nagelkerke R². The Hosmer–Lemeshow test result (χ² = 3.330, P = 0.912) suggested good model fit. The results of the hierarchical regression analysis are presented in Fig 6.

Except for change in exercise frequency weekly and grade (P > 0.05), all other variables remained in the model. The following factors were associated with a significantly increased risk of internet addiction among junior high school students: going to bed after 10:00 pm or having delayed sleep onset timing (OR = 2.859, 95% CI: 2.319–3.525); developing late-night eating habit (OR = 1.932, 95% CI: 1.494–2.499); becoming a smart device owner (OR = 1.773, 95% CI: 1.307–2.405); being a non-habitual napper (OR = 1.699, 95% CI: 1.408–2.049); decreased pursuit of dietary balance (OR = 1.654, 95% CI: 1.300–2.104); decreased active exercise (OR = 1.575, 95% CI: 1.222–2.031); and decrease in exercise duration per session (OR = 1.436, 95% CI: 1.117–1.846). Detailed results are shown in Fig 6.

### Model construction and predictive performance

**Fig 7:**
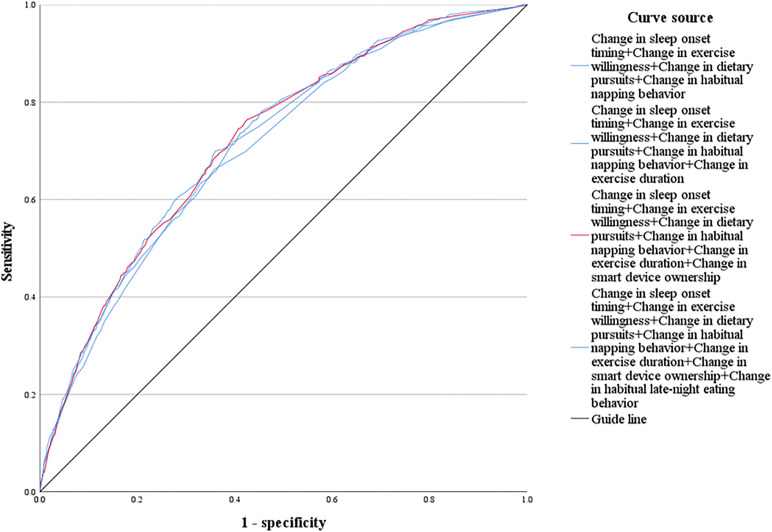
ROC curve.

Based on the predicted probability values derived from the classification variables and the true labels, Receiver Operating Characteristic (ROC) curves were plotted for predictive models incorporating different variables and their combinations. The results of the ROC curve analysis, presented in Table 2 and Fig 7, indicate that the predictive performance of the models improved as the number of variables increased. The seven-variable combined model achieved the highest Area Under the Curve (AUC) value of 0.723 (95% CI: 0.703–0.743), which was significantly superior to that of any single indicator (AUC range: 0.547–0.627) and other variable combinations (AUC range: 0.703–0.721). Detailed results are provided in Table 2 and Fig 7.

**Table 2:** Predictive Performance of Single and Combined Indicators for Internet Addiction Risk.

Variables	Optimal critical value	Sensitivity (%)	Specificity (%)	AUC (95%CI)
Predicted probability of change in sleep onset timing	0.084	80.60%	44.70%	0.627 (0.606 ～ 0.648)
Predicted probability of change in exercise willingness	0.081	49.70%	66.60%	0.596 (0.572 ～ 0.621)
Predicted probability of change in dietary pursuits	0.109	40.40%	76.10%	0.585 (0.561 ～ 0.609)
Predicted probability of change in habitual napping behavior	0.109	32.00%	80.50%	0.562 (0.538 ～ 0.587)
Predicted probability of change in exercise duration per session	0.079	79.50%	24.40%	0.554 (0.530 ～ 0.578)
Predicted probability of change in smart devices ownership	0.107	27.00%	82.50%	0.552 (0.528 ～ 0.576)
Predicted probability of change in habitual late-night eating behavior	0.101	18.00%	91.30%	0.547 (0.522 ～ 0.572)
Predicted probability of the combination of four variables	0.089	65.90%	64.40%	0.703 (0.682 ～ 0.724)
Predicted probability of the combination of five variables	0.075	72.40%	59.40%	0.712 (0.692 ～ 0.733)
Predicted probability of the combination of six variables	0.071	76.50%	57.40%	0.721 (0.701 ～ 0.741)
Predicted probability of the combination of seven variables	0.090	70.00%	63.90%	0.723 (0.703 ～ 0.743)

**Fig 8:**
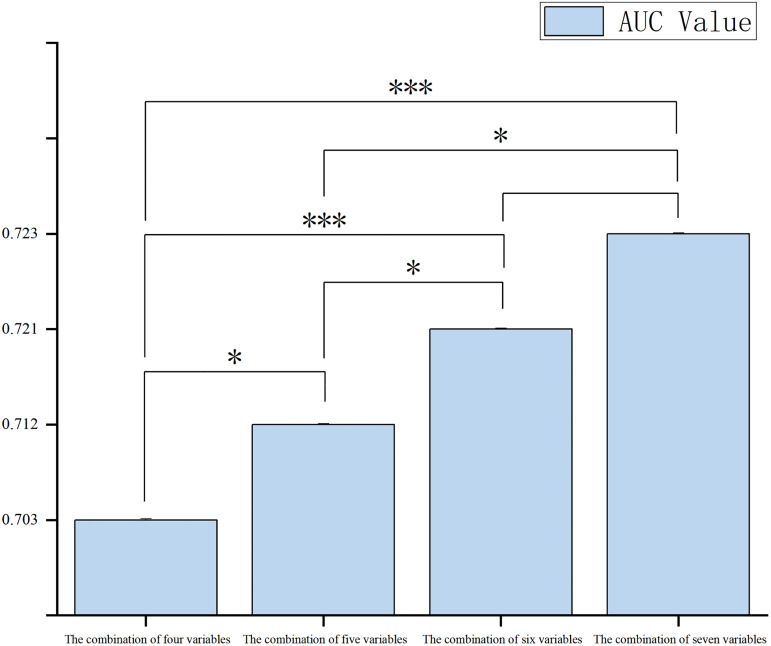
Results of the Delong test for comparison among the four ROC curves. *p < 0.05, **p < 0.01, ***p < 0.001.

After applying the DeLong test with Bonferroni correction, significant differences in AUC were observed among the models (overall *P* < 0.001). The seven-variable model did not differ significantly from the six-variable model (adjusted *P* > 0.05), but both were significantly superior to the five-variable and four-variable models (adjusted *P* < 0.05 for both comparisons). The six-variable model was selected as the optimal model, with an AUC of 0.721 (95% CI: 0.701–0.741). At the optimal cutoff value, the sensitivity was 76.50% and the specificity was 57.40%. The results of the model comparisons are shown in Fig 8.

## Discussion

In this study, the detection rate of Internet addiction risk among junior high school students increased from 10.62% at T1 to 13.35% at T2, which is consistent with recent domestic and international findings [6,28]. This study further examined how changes in the daily lifestyles of junior high school students may elevate the risk of Internet addiction. Through comparative analysis, we concluded that the following behavioral changes represent significant risk factors for Internet addiction: going to bed after 10:00 pm or having delayed sleep onset timing (OR = 2.859), developing a late-night eating habit (OR = 1.932), becoming a smart device owner (OR = 1.773), being a non-habitual napper (OR = 1.699), decreased pursuit of dietary balance (OR = 1.654), decreased active exercise (OR = 1.575), and decrease in exercise duration per session (OR = 1.436).

In the univariate analysis of Internet addiction status within the study groups (the non-internet addiction group and the newly established group), a significant difference was observed in the distribution of Internet addiction across different grades (*P* < 0.05). The proportion of students without Internet addiction declined progressively with higher grades, indicating that the junior high school stage remains a high-risk period for the development of Internet addiction. This finding aligns with the results reported by Onguner S. et al [29]. However, in the hierarchical regression model, grade was not a significant predictor of Internet addiction risk, suggesting that its effect may have been confounded or masked by other lifestyle variables included in the model. Similarly, change in exercise frequency weekly did not show a significant association with the dependent variable, implying that changes in exercise frequency alone may not be a key factor influencing susceptibility to Internet addiction among this population, or its effect may have been accounted for by more directly relevant variables such as change in exercise willingness and change in exercise duration per session in the model.

Furthermore, ROC curve analysis indicated that the six-variable lifestyle combination outperformed single variables and other variable combinations in predicting Internet addiction. Although the seven-variable model demonstrated the highest discriminative performance (AUC = 0.723), its difference from the six-variable model (AUC = 0.721) was not statistically significant (adjusted *P* > 0.05). Meanwhile, the six-variable model performed significantly better than models with fewer variables (all adjusted *P* < 0.05), suggesting that its predictive efficacy had reached a “plateau” comparable to that of the seven-variable model. According to the principle of parsimony, the six-variable model achieved an optimal balance between complexity and discriminative ability, making it more suitable for large-scale school-based screening.

At the optimal cutoff value (0.071), the six-variable model yielded a sensitivity of 76.50% and a specificity of 57.40%. This indicates that the model can correctly identify 76.50% of students at risk for Internet addiction, while correctly ruling out 57.40% of non-high-risk individuals. In school-based screening contexts, high sensitivity helps minimize missed cases, ensuring that most at-risk students are identified for further assessment. The moderate level of specificity implies a certain proportion of “false positives”—i.e., some non-addicted students may be misclassified as high-risk. This characteristic suggests that the model is better suited as an initial screening tool to identify individuals requiring subsequent professional evaluation, rather than serving as a definitive diagnostic instrument.

The six variables included in the model span four key dimensions: physical exercise, smart device ownership, diet, and sleep-wake patterns, which aligns closely with existing theoretical frameworks and empirical evidence.

Disrupted sleep-wake rhythm represents a core risk pathway. This study found that going to bed after 10:00 pm or having delayed sleep onset timing (OR = 2.859) had the strongest association with internet addiction risk. This may result from active late-night behaviors due to academic pressure [30], combined with melatonin suppression caused by blue light exposure from pre-sleep smart device use [31], both of which exacerbate circadian disruption. Sleep disturbances and high internet addiction risk often form a mutually reinforcing vicious cycle [32–34]. Notably, “delaying bedtime to use the internet” has become a significant link between internet addiction risk and sleep disorders [35]. Concurrently, establishing a regular napping habit also serves as an important factor in improving sleep quality [36]. Being a non-habitual napper (OR = 1.699) was also significantly associated with internet addiction risk. Regular napping may have a protective effect, potentially through mechanisms such as alleviating daytime fatigue, replenishing self-regulatory resources [37], and modulating the sensitivity of the brain’s reward system to internet-related stimuli [38].

Dysregulated eating behavior reflects an imbalance in lifestyle. Developing a late-night eating habit (OR = 1.932) and decreased pursuit of dietary balance (OR = 1.654) were both significantly associated with increased risk. Nighttime eating may disrupt circadian gene expression, lead to metabolic dysregulation, and, through gut–brain axis feedback mechanisms, heighten an individual’s drive for immediate rewards such as internet use. This can promote compensatory activation of the dopaminergic system in pursuit of stimulation (e.g., online gaming, short videos), forming a “metabolic–addiction cycle” [39]. Individuals addicted to the internet often further constrain their dietary habits, frequently skipping meals or consuming junk food and carbonated beverages while engaged online [14,40]. Unhealthy dietary patterns and internet addiction commonly coexist and mutually reinforce each other [41,42].

Accessibility to and supervision of smart device use are key environmental factors. Becoming a smart device owner (OR = 1.773) emerged as an independent risk factor, whose impact extends beyond increased exposure, potentially elevating addiction risk indirectly through mechanisms such as sleep disruption and substitution of real-world social interactions [43,44]. Effective parental supervision has been demonstrated as a protective factor [45,46]. While smart devices provide adolescents with convenient social channels, excessive reliance may contribute to the decline of real-life social skills [47]. Concurrently, platforms such as social media and online gaming are characterized by highly immediate feedback mechanisms, which readily stimulate dopamine release and can foster dependency on internet use [39,48]. This dependency may not only exacerbate internet addiction risk but can also interact with other mental health issues such as depression, anxiety, and attention-deficit disorders, forming a vicious cycle [44], thereby highlighting the importance of family-level intervention.

Diminished physical exercise weakens protective factors. Decreased active exercise (OR = 1.575) and decrease in exercise duration per session (OR = 1.436) were associated with increased risk. Existing research indicates that physical exercise significantly reduces the risk of internet addiction [49,50]. The underlying mechanisms include diverting attention away from the internet, modulating neurotransmitter systems such as dopamine, enhancing real-world social engagement and self-efficacy, thereby reducing dependency on the internet [39,51,52]. The present study did not identify an independent contribution of change in exercise frequency weekly, which may be attributable to the moderating effects of other variables or differences in measurement methods. However, studies have shown that individuals who engage in physical exercise 3–5 times per week exhibit a significantly lower risk of internet addiction compared to those with lower exercise frequency [53]. Meanwhile, other research suggests that the impact of exercise frequency and duration on internet addiction may vary across individuals depending on factors such as grit and self-control [54,55]. These discrepancies may be related to differences in the definitions and measurement approaches for exercise frequency and duration across studies, yet physical exercise remains widely recognized as a modifiable protective factor.

Taken together, these findings reveal a complex bidirectional relationship between internet addiction risk and unhealthy lifestyle factors—such as lack of exercise, irregular dietary patterns, and disrupted sleep—forming an interconnected and modifiable risk network [14,40,56].

Therefore, students, parents, and schools should actively attend to factors such as physical exercise, smart device ownership, dietary habits, and sleep-wake patterns that are associated with an elevated risk of internet addiction, thereby fostering a healthy lifestyle to prevent increased risk among junior high school students. In terms of diet, emphasis should be placed on balanced nutrition and reducing or avoiding late-night eating, helping students develop healthy eating habits. Regarding sleep-wake routines, parents should strengthen family supervision, particularly by limiting pre-bedtime mobile device use for internet access, assisting students in cultivating consistent and early bedtime habits, and appropriately arranging daytime naps. Concurrently, physical exercise should be enhanced; regular exercise not only improves physical health but also strengthens self-esteem and willpower, thereby increasing resilience to internet addiction [16]. Parents can allocate appropriate discretionary time for students to actively participate in cultural, sports, and social activities. Moreover, parents themselves should model positive behaviors [57] by reducing personal screen time, increasing companionship, strengthening supervision over students’ smart device use, and collaboratively establishing dynamic leisure activities that enrich real-world social engagement and reduce reliance on virtual social interaction and online entertainment.

This study has strengths in constructing a predictive model based on multidimensional lifestyle factors and identifying key modifiable targets, thereby providing empirical support for developing precise and comprehensive prevention strategies. However, several limitations should be noted. First, the questionnaire did not include details on napping and sleep (e.g., nap duration, timing of napping, nighttime sleep duration, sleep quality, and efficiency), specifics of physical exercise (e.g., intensity, types of exercise, and team-based activities), breakfast consumption, or systematic measures of anxiety, depression, insomnia, or other psychological comorbidities [58,59], which may have led to the omission of important covariates. Second, daily lifestyle factors were assessed using a self-designed questionnaire with responses based on self-report, which may be subject to recall bias and subjective bias. Additionally, as the respondents were minors, even under anonymous conditions, they may have underreported undesirable lifestyle behaviors, potentially leading to an underestimation of effects and limiting the generalizability of the findings. Third, although the six-variable model achieved an AUC of 0.721, outperforming single indicators and other combinations, there remains room for improvement, suggesting that clinical interviews and multi-source information should be integrated for comprehensive assessment. Fourth, the sample was limited to junior high school students from one district in Jingzhou City; future studies should expand the sampling scope to enhance representativeness. Fifth, the availability of only two waves of data constrained a more comprehensive understanding of the dynamic changes in the variables over time. Future research should consider longer time spans and continued longitudinal follow-up.

## Conclusions

In summary, this study confirms that a composite model based on multidimensional lifestyle indicators demonstrates good predictive performance for internet addiction among junior high school students. The six-variable model achieved an optimal balance between discriminative efficacy and practical feasibility, making it suitable for use as a preliminary screening tool. Lack of exercise, excessive smart device use, sleep disturbances, and dysregulated eating can serve as core intervention targets. Future interventions should integrate efforts across school, family, and individual levels, focusing on these modifiable lifestyle factors to effectively prevent and reduce the risk of internet addiction among junior high school students.

## References

[1] Young KS. Internet Addiction: The Emergence of a New Clinical Disorder. CyberPsychology & Behavior. 1998;1(3):237–44. doi: 10.1089/cpb.1998.1.237

[2] Block JJ. Issues for DSM-V: internet addiction. Am J Psychiatry. 2008;165(3):306–7. doi: 10.1176/appi.ajp.2007.07101556 18316427

[3] Ernst M, Pine DS, Hardin M. Triadic model of the neurobiology of motivated behavior in adolescence. Psychol Med. 2006;36(3):299–312. doi: 10.1017/S0033291705005891 16472412 PMC2733162

[4] Israelashvili M, Kim T, Bukobza G. Adolescents’ over-use of the cyber world--Internet addiction or identity exploration? J Adolesc. 2012;35(2):417–24. doi: 10.1016/j.adolescence.2011.07.015 21803411

[5] Blinka L, Škařupová K, Ševčíková A, Wölfling K, Müller KW, Dreier M. Excessive internet use in European adolescents: what determines differences in severity? Int J Public Health. 2015;60(2):249–56. doi: 10.1007/s00038-014-0635-x 25532555

[6] Mak K-K, Lai C-M, Watanabe H, Kim D-I, Bahar N, Ramos M, et al. Epidemiology of internet behaviors and addiction among adolescents in six Asian countries. Cyberpsychol Behav Soc Netw. 2014;17(11):720–8. doi: 10.1089/cyber.2014.0139 25405785

[7] Ha JH, Yoo HJ, Cho IH, Chin B, Shin D, Kim JH. Psychiatric comorbidity assessed in Korean children and adolescents who screen positive for Internet addiction. J Clin Psychiatry. 2006;67(5):821–6. doi: 10.4088/jcp.v67n0517 16841632

[8] Tran BX, Huong LT, Hinh ND, Nguyen LH, Le BN, Nong VM, et al. A study on the influence of internet addiction and online interpersonal influences on health-related quality of life in young Vietnamese. BMC Public Health. 2017;17(1):138. doi: 10.1186/s12889-016-3983-z 28143462 PMC5282902

[9] Moreno MA, Eickhoff J, Zhao Q, Young HN, Cox ED. Problematic Internet Use: A longitudinal study evaluating prevalence and predictors. J Pediatr X. 2019;1:100006. doi: 10.1016/j.ympdx.2019.100006 34308328 PMC8300087

[10] Güell F, Núnez L. The liberating dimension of human habit in addiction context. Front Hum Neurosci. 2014;8:664. doi: 10.3389/fnhum.2014.00664 25221498 PMC4147296

[11] Ramírez-Vizcaya S, Froese T. The Enactive Approach to Habits: New Concepts for the Cognitive Science of Bad Habits and Addiction. Front Psychol. 2019;10:301. doi: 10.3389/fpsyg.2019.00301 30863334 PMC6399396

[12] Di Paolo E, Buhrmann T, Barandiaran X. Sensorimotor Life: An Enactive Proposal. Oxford University Press; 2017.

[13] Davis RA. A cognitive-behavioral model of pathological Internet use. Computers in Human Behavior. 2001;17(2):187–95. doi: 10.1016/s0747-5632(00)00041-8

[14] Shokri A, Mohamadi A, Mohammadi D, Moradi M, Sadeghi S, Mahmoodi H, et al. The relationship between internet addiction and lifestyle among high school students: A cross sectional in the west of Iran. PLoS One. 2024;19(9):e0308333. doi: 10.1371/journal.pone.0308333 39240897 PMC11379132

[15] Lianov L, Johnson M. Physician competencies for prescribing lifestyle medicine. JAMA. 2010;304(2):202–3. doi: 10.1001/jama.2010.903 20628134

[16] Gu H, Shi B, He H, Yuan S, Cai J, Chen X, et al. Association between excessive internet use time, internet addiction, and physical-mental multimorbidity among Chinese adolescents: Cross-sectional study. J Med Internet Res. 2025;27(e69210). 10.2196/69210 PMC12138303 40397924

[17] Roehrick KC, Vaid SS, Harari GM. Situating smartphones in daily life: Big Five traits and contexts associated with young adults’ smartphone use. J Pers Soc Psychol. 2023;125(5):1096–118. doi: 10.1037/pspp0000478 37956069

[18] Schermer EE, Engelfriet PM, Blokstra A, Verschuren WMM, Picavet HSJ. Healthy lifestyle over the life course: Population trends and individual changes over 30 years of the Doetinchem Cohort Study. Front Public Health. 2022;10:966155. doi: 10.3389/fpubh.2022.966155 36159268 PMC9500162

[19] Gardner B. A review and analysis of the use of “habit” in understanding, predicting and influencing health-related behaviour. Health Psychol Rev. 2015;9(3):277–95. doi: 10.1080/17437199.2013.876238 25207647 PMC4566897

[20] Kim Y, Park JY, Kim SB, Jung I-K, Lim YS, Kim J-H. The effects of Internet addiction on the lifestyle and dietary behavior of Korean adolescents. Nutr Res Pract. 2010;4(1):51–7. doi: 10.4162/nrp.2010.4.1.51 20198209 PMC2830415

[21] Zhong X, Li M, Li L. Preventing and detecting insufficient effort survey responding. Adv Psychol Sci. 2021;29(2):225–37. doi: 10.3724/sp.j.1042.2021.00225

[22] Liu C, Zhou L, Pi X-X, Liu B, Zhang X-F, Wei W-C, et al. Network characteristics of the youth’s insomnia and emotional symptoms and their gender differences. Front Psychiatry. 2025;16:1597652. doi: 10.3389/fpsyt.2025.1597652 40589648 PMC12206766

[23] Moon SJ, Hwang JS, Kim JY, Shin AL, Bae SM, Kim JW. Psychometric properties of the Internet Addiction Test: A systematic review and meta-analysis. Cyberpsychology, Behavior, and Social Networking. 2018;21(8):473–84. 30110200 10.1089/cyber.2018.0154

[24] Hirota T, McElroy E, So R. Network Analysis of Internet Addiction Symptoms Among a Clinical Sample of Japanese Adolescents with Autism Spectrum Disorder. J Autism Dev Disord. 2021;51(8):2764–72. doi: 10.1007/s10803-020-04714-x 33040268

[25] Lu J, Zhang Q, Zhong N, Chen J, Zhai Y, Guo L, et al. Addiction Symptom Network of Young Internet Users: Network Analysis. J Med Internet Res. 2022;24(11):e38984. doi: 10.2196/38984 36355402 PMC9693725

[26] Carter JV, Pan J, Rai SN, Galandiuk S. ROC-ing along: Evaluation and interpretation of receiver operating characteristic curves. Surgery. 2016;159(6):1638–45. doi: 10.1016/j.surg.2015.12.029 26962006

[27] Gorman SL, Radtka S, Melnick ME, Abrams GM, Byl NN. Development and validation of the Function In Sitting Test in adults with acute stroke. J Neurol Phys Ther. 2010;34(3):150–60. doi: 10.1097/NPT.0b013e3181f0065f 20716989

[28] Yoo Y-S, Cho O-H, Cha K-S. Associations between overuse of the internet and mental health in adolescents. Nurs Health Sci. 2014;16(2):193–200. doi: 10.1111/nhs.12086 23991723

[29] Onguner S, Şahin Ş, Akçaboy M, Altınel Açoğlu E, Oğuz MM, Yücel H, et al. Internet Addiction of School-Age Children and the Effects of Daily Habits. Cyprus Journal of Medical Sciences. 2024:241–8. doi: 10.4274/cjms.2024.2022-23

[30] Mehta KJ. Effect of sleep and mood on academic performance—at interface of physiology, psychology, and education. Humanit Soc Sci Commun. 2022;9(1). doi: 10.1057/s41599-021-01031-1

[31] Wood B, Rea MS, Plitnick B, Figueiro MG. Light level and duration of exposure determine the impact of self-luminous tablets on melatonin suppression. Appl Ergon. 2013;44(2):237–40. doi: 10.1016/j.apergo.2012.07.008 22850476

[32] Touitou Y, Touitou D, Reinberg A. Disruption of adolescents’ circadian clock: The vicious circle of media use, exposure to light at night, sleep loss and risk behaviors. J Physiol Paris. 2016;110(4 Pt B):467–79. doi: 10.1016/j.jphysparis.2017.05.001 28487255

[33] Chang A-M, Aeschbach D, Duffy JF, Czeisler CA. Evening use of light-emitting eReaders negatively affects sleep, circadian timing, and next-morning alertness. Proc Natl Acad Sci U S A. 2015;112(4):1232–7. doi: 10.1073/pnas.1418490112 25535358 PMC4313820

[34] Alimoradi Z, Lin C-Y, Broström A, Bülow PH, Bajalan Z, Griffiths MD, et al. Internet addiction and sleep problems: A systematic review and meta-analysis. Sleep Med Rev. 2019;47:51–61. doi: 10.1016/j.smrv.2019.06.004 31336284

[35] Lu JX, Zhai YJ, Chen J, Zhang QH, Chen TZ, Lu CL. Network analysis of internet addiction and sleep disturbance symptoms. Progress in Neuro-Psychopharmacology & Biological Psychiatry. 2023;125:110737. 36868497 10.1016/j.pnpbp.2023.110737

[36] Gupta R, Taneja N, Anand T, Gupta A, Gupta R, Jha D. Internet addiction, sleep quality and depressive symptoms amongst medical students in Delhi, India. Community Mental Health Journal. 2020;57(4):771–6. 32852657 10.1007/s10597-020-00697-2

[37] Baumeister RF, Bratslavsky E, Muraven M, Tice DM. Ego depletion: is the active self a limited resource? Journal of Personality and Social Psychology. 1998;74(5):1252–65. 9599441 10.1037//0022-3514.74.5.1252

[38] Brand M. Can internet use become addictive? Science. 2022;376(6595):798–9. 35587961 10.1126/science.abn4189

[39] Li Y, Ma J, Yao K, Su W, Tan B, Wu X, et al. Circadian rhythms and obesity: Timekeeping governs lipid metabolism. J Pineal Res. 2020;69(3):e12682. doi: 10.1111/jpi.12682 32656907

[40] Radwan H, Abdelrahim DN, Osaili T, Thabet Y, Barakat H, Khetrish M, et al. The association of binge eating with internet addiction, body shape concerns, and BMI among university students in the United Arab Emirates. J Eat Disord. 2025;13(1):21. doi: 10.1186/s40337-025-01205-1 39930515 PMC11809045

[41] Tayhan Kartal F, Yabancı Ayhan N. Relationship between eating disorders and internet and smartphone addiction in college students. Eat Weight Disord. 2021;26(6):1853–62. doi: 10.1007/s40519-020-01027-x 33034868

[42] Tao Z, Wu G, Wang Z. The relationship between high residential density in student dormitories and anxiety, binge eating and Internet addiction: a study of Chinese college students. Springerplus. 2016;5(1):1579. doi: 10.1186/s40064-016-3246-6 27652152 PMC5025399

[43] Paulus MP, Squeglia LM, Bagot K, Jacobus J, Kuplicki R, Breslin FJ, et al. Screen media activity and brain structure in youth: Evidence for diverse structural correlation networks from the ABCD study. Neuroimage. 2019;185:140–53. doi: 10.1016/j.neuroimage.2018.10.040 30339913 PMC6487868

[44] Wacks Y, Weinstein AM. Excessive Smartphone Use Is Associated With Health Problems in Adolescents and Young Adults. Front Psychiatry. 2021;12:669042. doi: 10.3389/fpsyt.2021.669042 34140904 PMC8204720

[45] Meng Z, Min K, Ma R, Yang J, Zhang H, Li Q. The mediating effect of parental monitoring in the association between parent-child relationship harmony and smartphone addiction: findings from a nationwide youth survey in China. BMC Public Health. 2025;25(1):1184. doi: 10.1186/s12889-025-22366-3 40155835 PMC11951532

[46] Tom A, Thomas B, Sharma M, Joseph A. Parental rejection and control: Potential risks for excessive Internet usage among adolescents. Int J Soc Psychiatry. 2023;69(8):2007–17. doi: 10.1177/00207640231185450 37539691

[47] Yoon S, Lee NY, Hong S. Problematic smartphone use, online classes, and middle school students’ social-emotional competencies during COVID 19: mediation by lifestyle and peers. Humanit Soc Sci Commun. 2025;12(1). doi: 10.1057/s41599-025-04689-z

[48] Gao W, Ping S, Liu X. Gender differences in depression, anxiety, and stress among college students: A longitudinal study from China. J Affect Disord. 2020;263:292–300. doi: 10.1016/j.jad.2019.11.121 31818792

[49] Cai P, Wang J, Ye P, Feng X, Yang G, Huang C, et al. Physical exercise/sports ameliorate the internet addiction from college students during the pandemic of COVID-19 in China. Front Public Health. 2023;11:1310213. doi: 10.3389/fpubh.2023.1310213 38179571 PMC10764417

[50] Sönmez Sari E, Terzi H, Şahin D. Social media addiction and cognitive behavioral physical activity among adolescent girls: A cross-sectional study. Public Health Nursing. 2025;42(1):61–9. 39402902 10.1111/phn.13446 PMC11700928

[51] Xu J, Tang L. The relationship between physical exercise and problematic internet use in college students: the chain-mediated role of self-control and loneliness. BMC Public Health. 2024;24(1):1719. doi: 10.1186/s12889-024-19226-x 38937729 PMC11212378

[52] Li S, Wu Q, Tang C, Chen Z, Liu L. Exercise-Based Interventions for Internet Addiction: Neurobiological and Neuropsychological Evidence. Front Psychol. 2020;11:1296. doi: 10.3389/fpsyg.2020.01296 32670157 PMC7330165

[53] Zhihao D, Tao W, Yingjie S, Feng Z. The influence of physical activity on internet addiction among Chinese college students: the mediating role of self-esteem and the moderating role of gender. BMC Public Health. 2024;24(1):935. doi: 10.1186/s12889-024-18474-1 38561700 PMC10986089

[54] Luo M, Duan Z, Chen X. The role of physical activity in mitigating stress-induced internet addiction among Chinese college students. J Affect Disord. 2024;366:459–65. doi: 10.1016/j.jad.2024.08.188 39216640

[55] Li X, Wang J, Yu H, Liu Y, Xu X, Lin J, et al. How does physical activity improve adolescent resilience? Serial indirect effects via self-efficacy and basic psychological needs. PeerJ. 2024;12:e17059. doi: 10.7717/peerj.17059 38436018 PMC10909365

[56] Arafa A, Yasui Y, Kokubo Y, Kato Y, Matsumoto C, Teramoto M, et al. Lifestyle Behaviors of Childhood and Adolescence: Contributing Factors, Health Consequences, and Potential Interventions. Am J Lifestyle Med. 2024:15598276241245941. doi: 10.1177/15598276241245941 39554934 PMC11562273

[57] Cheng L, Cao J. Factors influencing smart device addiction among preschool children: An extended protection-risk model perspective. Front Psychol. 2023;14:1017772. doi: 10.3389/fpsyg.2023.1017772 36844311 PMC9947858

[58] Yang X, Guo W-J, Tao Y-J, Meng Y-J, Wang H-Y, Li X-J, et al. A bidirectional association between internet addiction and depression: A large-sample longitudinal study among Chinese university students. J Affect Disord. 2022;299:416–24. doi: 10.1016/j.jad.2021.12.013 34906641

[59] Wolff JC, Thompson E, Thomas SA, Nesi J, Bettis AH, Ransford B, et al. Emotion dysregulation and non-suicidal self-injury: A systematic review and meta-analysis. Eur Psychiatry. 2019;59:25–36. doi: 10.1016/j.eurpsy.2019.03.004 30986729 PMC6538442

